# A Gene Expression Study of the Activities of Aromatic Ring-Cleavage Dioxygenases in *Mycobacterium gilvum* PYR-GCK to Changes in Salinity and pH during Pyrene Degradation

**DOI:** 10.1371/journal.pone.0058066

**Published:** 2013-02-28

**Authors:** Abimbola Comfort Badejo, Adegoke Olugboyega Badejo, Kyung Hoon Shin, Young Gyu Chai

**Affiliations:** 1 Department of Molecular and Life Sciences, Hanyang University, Ansan, Korea; 2 Department of Environmental and Marine Science, Hanyang University, Ansan, Korea; University of Kansas, United States of America

## Abstract

Polycyclic aromatic hydrocarbons (PAHs) are toxic pollutants found in the environment which can be removed through the use of physical and biological agents. The rate of PAH biodegradation is affected by environmental conditions of pH, salinity and temperature. Adaptation of the pyrene degrading bacteria, *Mycobacterium gilvum* PYR-GCK, to fluctuating environmental conditions during pyrene biodegrading activity was studied using the quantitative real time – Polymerase Chain Reaction (qRT-PCR) technique. Four aromatic ring-cleavage dioxygenase genes: *phdF, phdI*, *pcaG* and *pcaH*; critical to pyrene biodegradation, were studied in pH states of 5.5, 6.5, 7.5 and NaCl concentrations 0 M, 0.17 M, 0.5 M, 0.6 M, 1 M. First, we conducted a residual pyrene study using gas chromatography and flame ionization technologies. Central to a gene expression study is the use of a valid endogenous reference gene, making its determination our next approach, using the geNorm/NormFinder algorithms. Armed with a valid control gene, *rpoB*, we applied it to a gene expression study, using the comparative critical threshold (2^ΔΔCT^) quantification method. The pyrene degrading activity of the strain was strongly functional in all the NaCl concentration states, with the least activity found at 1M (∼70% degraded after 48 hours of cultivation). The transcripts quantification of three genes backed this observation with high expression levels. The gene expression levels also revealed pH 6.5 as optimal for pyrene degradation and weak degradation activity at pH of 5.5, corroborating the residual pyrene analysis. The expression of these genes as proteins has already been studied in our laboratory using proteomics techniques and this validates our current study.

## Introduction

Polycyclic aromatic hydrocarbons (PAHs) are components of incomplete combustion of fossil fuels released into the environment by natural or anthropogenic means [1]. Their hydrophobic states and persistence in the environment makes them toxic to living organisms by eliciting mutagenic or carcinogenic responses [2], [3]. Some of these PAHs have been listed by the US Environmental Protection Agency (EPA) as the top 16 PAH priority pollutants [4] and their effects being studied worldwide with pyrene as a model compound due to its structural similarity to several carcinogenic PAHs [5], [6], [7]. Microorganisms (bacteria and fungi) can mineralize some of these PAHs by transforming them into molecules that can enter into their central metabolic pathways. An example of such a microorganism is the *Mycobacterium* sp. which has been under study due to its ability to utilize numerous PAHs as sole sources of carbon and energy. Its biodegradative ability is being studied as a feasible bioremediation technology application for hazardous organic pollutants [8], [9], [10].

Reports on the effects of various environmental factors such as pH, temperature and salinity on the biodegradation rates of PAHs in the environment have been documented [11], [12]. One report has shown that a pH shift from 5.2 to 7.0 induced a ten-fold increase in PAH biodegrading activity in bacteria inoculated soils [12] while another showed that the acidification (pH 6.5) of a mycobacterial culture resulted in a four-fold rate increment of pyrene and phenanthrene degradation; which might have been as a result of a more permeable cell membrane to the PAH hydrophobic molecules [11]. Diaz and his colleagues [13] recorded in their research that crude oil biodegradation was greater at lower salinity and decreased at salinities twice that of normal sea water (35 g/L) while *Mycobacterium smegmatis* have been observed to survive in cultures with salinity concentrations as high as 1 M NaCl [14]. The ocean and terrestrial environments are susceptible to PAH pollution from oil-drilling (on/off-shore), crude oil refining process and tanker spills; and industrial waste effluents. These polluted habitats have varying conditions of pH and salinity as a result of processes such as ocean acidification, soil acidification and industrial waste effluent treatments [15], [16], [17], [18]. Bacterial bioremediation of these contaminated environments is feasible provided the applied bioremediation technology is compatible.

The multiple findings of the various effects of pH and salinity concentrations have prompted a molecular research on gene expression, focusing on the activities of some key genes/enzymes in pyrene degradation as a result of acidification and different NaCl concentrations induction. An insight into the molecular adaptation of the mycobacterial strain to the various states of pH and salinity concentrations, during pyrene biodegradation, will help in the development of potential bioremediation applications.

Aerobic bacterial biodegradation of aromatic compounds employ the use of many enzymes which include various dioxygenases and dehydrogenases [19]. Central to PAH degradation processes is the opening of the thermodynamically stable benzene rings by aromatic ring cleaving dioxygenases (ARCDs) [20], [21], [22]. The focus of this research was based on the expression activities of ARCD genes namely: phdF (coding for an extradiol dioxygenase), phdI (coding for 1-hydroxy-2-naphthoate dioxygenase/gentisate-1,2-dioxygenase), pcaG and H (coding for the alpha and beta subunits of protocatechuate-3,4-dioxygenase respectively). These genes were positively expressed in the bacteria *Mycobacterium gilvum* PYR-GCK, in response to pyrene induction in a previous proteomics study [20]. Extradiol dioxygenase has been proposed to catalyze the conversion of the four-ringed dihydrodiol: 4,5-dihydroxypyrene, and the three-ringed dihydrodiol: 3,4-dihydroxyphenanthrene into their lesser ringed carboxylate counterparts in the pyrene degradation pathway [23], [24] while 1-hydroxy-2-naphthoate dioxygenase cleaves a singly hydroxylated aromatic ring present in 1-hydroxy-2-naphthoate to produce trans-2-carboxy benzal pyruvate [25], [26]. Protocatechuate 3,4-dioxygenase enzyme subunits catalyze protocatechuic acid cleavage and not catechol in Streptomyces sp. strain 2065 [27], breaking the final aromatic substrate ring into β-carboxy- cis, cis-muconate and subsequently releasing the pyrene degraded intermediates into the central metabolic pathway [23], [25], [27].

*Mycobacterium gilvum* PYR-GCK (ATCC 700033), isolated from the sediment of the Grand Calumet River in Northwestern Indiana based on its ability to utilize pyrene as a growth substrate [28], was used for this research due to the availability of necessary information such as its genome annotations (http://jgi.doe.gov/) and expressed proteins as a result of pyrene induction. This has provided a foundation for further studies on the strain's biodegradative activity in different environmental conditions; to give vital information on the development of future bioremediation applications.

## Materials and Methods

### Reagents and bacterial strain maintenance

*Mycobacterium gilvum* PYR-GCK was acquired from the American Type Culture Collection under the code name *Mycobacterium flavescens* ATCC 700033. The strain was maintained in Bacto Brain Heart Infusion agar plates (BD Laboratories, Sparks, USA) at 29°C or stock preserved in same media (broth) supplemented with 28% glycerol at 80°C. The pyrene substrate (confirmed >98.0% pure by Aldrich Company) and other chemicals used were purchased from Sigma-Aldrich Company (St. Louis, USA) and Tokyo Chemical Industry (Tokyo, Japan).

### Growth media and strain cultivation

*M.gilvum* PYR-GCK cells were grown in 500 ml flasks of 300 ml basal medium containing, per litre: NaNO_3_, 0.5 g; (NH_4_)_2_SO_4_, 1.0 g; Na_2_HPO_4_; 2.5 g; KH_2_PO_4_, 1.0 g; MgSO_4_•7H_2_O, 0.1 g; Fe(NH_4_)_2_(SO_4_)_2_, 5 mg; 1 ml filter-sterilized Vitamin solution (containing, per litre: p-aminobenzoic acid, 200 mg; biotin, 200 mg; folic acid, 200 mg; nicotinic acid, 200 mg; Ca-panthothenate, 100 mg; pyridoxine-HCl, 100 mg; riboflavin, 100 mg; thiamine, 100 mg and vitamin B12, 1 mg) and 1 ml Trace Elements solution (containing, per litre: MnCl_2_•2H_2_O, 23 mg; H_3_BO_3_, 31 mg; CoCl_2_ 6H_2_O, 36 mg; CuCl_2_•2H_2_O, 10 mg; NiCl_2_ 6H_2_O, 20 mg; ZnCl_2_, 50 mg and Na_2_MoO_4_•2H_2_O, 30 mg) sterilized separately. The pH of the various culture flasks were adjusted to 5.5, 6.5 and 7.5, at zero salinity. Pyrene was dissolved in dimethyl sulfoxide and added to the induced culture-flasks at a final concentration of 25 µM while the control-culture flask had no substrate added and pH adjusted to 7.0. Cells (OD_545_2.956) were inoculated into the culture flasks after being washed twice in 50 mM monobasic sodium phosphate buffer solutions of their respective pHs 5.5, 6.5 and 7.5. For the salinity experiments, the media and the respective monobasic sodium phosphate washing buffer solutions were adjusted to salinities of 0 M, 0.17 M, 0.5 M, 0.6 M and 1 M (representing 0 g/L, 1 g/L, 29 g/L, 35 g/L and 58 g/L respectively) and pH 7.0. All cultures were prepared in duplicates and incubated at 30°C with shaking at 150 rpm for 48 hours in the dark.

### Pyrene utilization at the various growth conditions

Pyrene substrate extraction from the pyrene-induced cells and culture, were carried out as described in [20], [29]. The extraction procedure was enhanced by sonication at 200W for 5 mins at 30∶15 seconds pulse on ice. After evaporation to dryness in the rotatory evaporator, the dried residue was reconstituted in a small volume of acetonitrile, filtered in a glass syringe fitted with a 0.2 µl Teflon Membrane filter (Millipore, Bedford, USA) into 1.5 ml amber High recovery Screw top vials (Agilent, Santa Clara, USA). The extracts were concentrated to 1 ml with nitrogen gas after which 200 µl of the extracts is reacted with 100 µl of N, O-Bis (trimethylsilyl) trifluoroacetamide (BSTFA) at 68°C for 1 hour. The silylated extracts were further diluted down with 600 µl acetonitrile before loading the vials on the GC/MS instrument (GCMS-QP2010; Shimadzu, Kyoto, Japan) via the auto- sampler. Quantification of residual pyrene for the growth experiments was performed using GC/MS coupled with FID with a J&W DB-5 capillary (30 m×0.25 mm diameter), programmed from 50°C to 300°C at a rate of 6°C/min and held at 300°C for 10 mins. The carrier gas used was helium. Quantification was achieved by integration of FID peak areas; 2-nonadecanone was used as a reference (injection) standard.

### Total RNA extraction

Zero-, 12-, 24-, 36- and 48-hour-old cells were harvested from broth by centrifugation at 10,000× *g* at 4°C for 1 min, after the addition of RNAprotect Bacteria reagent (Qiagen, California, USA) to the culture broth in the ratio 2∶1. RNAiso (Takara, Japan) lysing solution was added to the cells, along with 10 µl β-mercaptoethanol and 0.6 g of 0.1 mm Zirconia/Silica beads (Biospec, Oklahoma, USA). The mix was run in mini Bead-beater (Biospec, Oklahoma, USA) for 45 seconds and immediately placed on ice. Two hundred microliters of chloroform was added to the solution and the tubes with the lysing mix were inverted gently to mix for 5 minutes. The mix was centrifuged at 12,000× *g* for 15 mins at 4°C and the clear top solution was carefully collected into a new tube. Five hundred microliters isopropanol was added and the tubes were gently inverted to mix once again before it was finally incubated on ice for 1 hour. After incubation, the lysed mix was centrifuged at 12,000× *g* for 10 mins at 4°C and the isopropanol was decanted. Ice-cold 70% ethanol was added to the RNA pellet for gentle washing. After another round of centrifuging at same speed for 10 mins, the ethanol was carefully removed. RNA pellets were left to dry at room temperature for 5–10 minutes before reconstitution in 20 µl RNase-free water. The RNA was treated with RNase-free DNase (Promega, Wisconsin, USA) and purified by extraction with phenol: chloroform: isoamyl alcohol (25∶24∶1). The concentration of the purified RNA was determined by using a Nanodrop ND-1000 spectrophotometer (Nanodrop Technologies, Delaware, USA).

### cDNA synthesis and gene expression quantification

Reverse transcription of RNA samples were performed as described by Kim et al. [30] using 2 µg of total RNA sample, 1 µl random hexamers (per reaction) and PrimeScript 1st strand cDNA synthesis Kit (Takara, Japan). Briefly, random hexamers and RNA templates were mixed and denatured at 65°C for 5 min followed by cooling for 2 min on ice. Primescript buffer (5×), RTase and RNAse inhibitor were added to the cooled template mix and incubated for 1 hour at 50°C before enzyme inactivation at 70°C for 15 min. Negative controls for reverse transcription were performed to test for the presence of genomic DNA contamination in the RNA samples. Complementary DNA samples were diluted 1.5-fold and a quantitative real-time PCR run was employed on AB-7500 Real-time thermal cycler (Applied Biosystems, California, USA) using SYBR Premix Ex-Taq II (Takara Bio, Shiga, Japan) according to manufacturer's directions. PCR volume was 20 µl consisting of 0.4 µM of each primer (Table 1). Each PCR run included a no-template control with water instead of cDNA as well as a RT negative control for each gene. Triplicate measurements were performed for all reactions. pH and NaCl concentration samples were carried on separate 96-well plates for the gene expression experiments while all samples were analyzed on a single plate for the endogenous control determination experiment. Results were analyzed using the critical threshold (ΔC_T_) and the comparative critical threshold (ΔΔC_T_) method in the AB-7500 software, the Normfinder and the geNorm-plus algorithms. (Primer Design, Southampton, UK).

**Table 1:** Primers used in qRT-PCR studies of pH and salinity changes- induced pyrene degradation and the reference genes of *Mycobacterium gilvum* PYR-GCK.

Primer sequences		
Gene designation	Forward (5′–3′)	Reverse (5′–3′)
*rrs*	GGCGTGCTTAACACATGCAA	GCATGCGGTCCTATTCGGTA
*rpoB*	GTCATCGTCTGGTCACCCTG	AGGTCAACAAGAAGCTCGG
*rpoD*	GTCGCTCCGGGACCACATCC	TCAGGAGGTGTGCTTGGCCG
*dnaG*	TCACCACCGGCGTCCAGTCT	CCCTGCTCGACGGGGGACAT
*phdF*	GCACCACCTTCTGACCGTAA	TTGGGTTTGAGGTGGGAACC
*phdI*	TGACGAAGTGATGGGTGCTC	AGTGCCGTGTATTTCGTCGT
*pcaG*	GGTGTCCTGCAGTTGGATGT	TACATTCCCGGCAAGCAGTT
*pcaH*	GTTGAGACTGGCGAACGGTA	AATGTTCAGCAAACGCGAGG

Genes description and locus tag are as follows: **Candidate endogenous control genes**: *rrs* (16S RNA ribosomal subunit: Mflv_ R0023), *rpoB* (DNA-directed RNA polymerase subunit: Mflv_5097), *rpoD* (RNA polymerase subunit, sigma-70 family: Mflv_4912), *dnaG* (Primase: Mflv_2722); **Aromatic ring-cleaving dioxygenase genes of interest:** *phdF* (Extradiol dioxygenase: Mflv_ 0538), phdI (1-hydroxy-2-naphthoate dioxygenase/gentisate-1,2-dioxygenase: Mflv_ 0589), pcaG (protocatechuate-3,4-dioxygenase, alpha subunit: Mflv_0529), pcaH (protocatechuate-3,4-dioxygenase, beta subunit: Mflv_ 0530). All primers were generated using Primer Express software (version 2.0).

## Results

### Pyrene degradation in the various pH and NaCl concentrations

**Figure 1:**
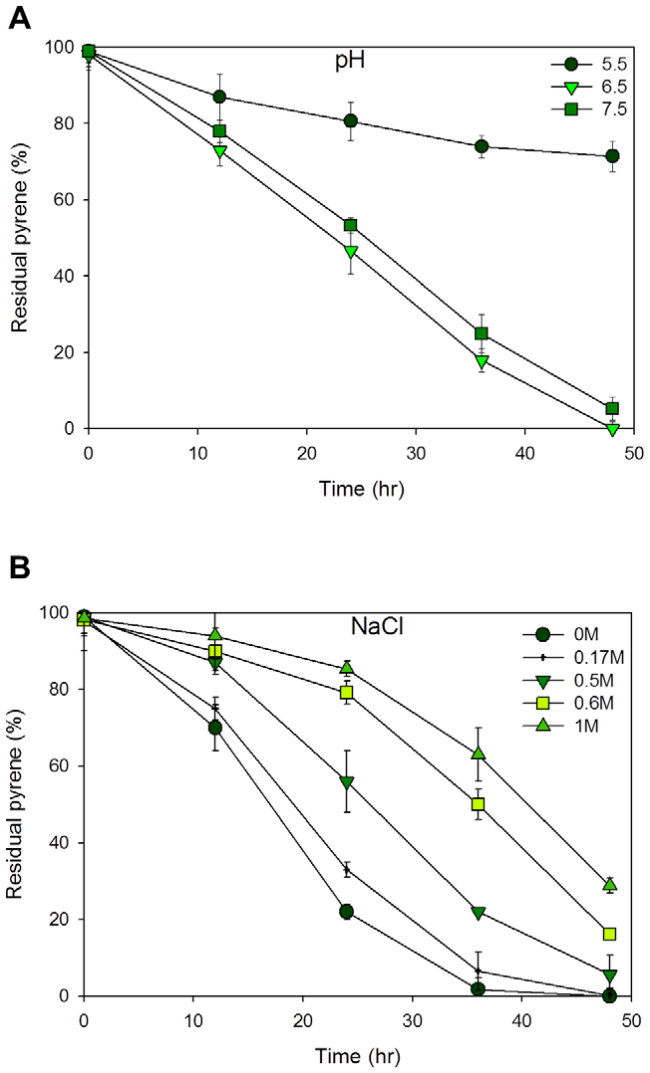
Pyrene degradation profiles showing the residual pyrene (%) in the various cultures. Graph of culture induced with pH states of 5.5, 6.5 and 7.5 (A) and NaCl concentrations of 0 M, 0.17 M, 0.5, 0.6 and 1 M (B). pH states correspond to acidic nature of the oceans and polluted terrestrial environments while the NaCl concentrations correspond to the saline nature of the ocean and some industrial waste effluents. Data and standard error are means from two replicates.

At various times during bacterial growth/induction, 10 ml samples were extracted for residual pyrene determination. Fig. 1A of the residual pyrene profile shows reduced pyrene degradation activity at pH 5.5 with 30% of total pyrene degraded after 48 hours of cultivation. Extreme degradation activities were observed at the 48th hour of cultivation in the pH 6.5 and 7.5 cultures. The highest degradation activity was recorded in the pH 6.5 culture with zero residual pyrene after 58 hours. This makes a slightly acidic condition, probably, the best condition for pyrene degradation in our experimental strain.

The salinity conditions conferred to the cultures showed results highlighting the halotolerant nature of the strain at our experimental conditions (Fig. 1B). High degree of pyrene degrading activities were observed in the 0 M and 0.17 M induced cultures with approximately zero pyrene left at 48 hour, in the flasks. Degradation at 0.5 M NaCl concentration was slightly of a lower rate with 5.6% pyrene left at 48th hour of cultivation. Slowest degradation rates were observed in the 0.6 M and 1 M NaCl cultures with 16.2% and 28.8% pyrene left at the 48th hour of cultivation.

### Determining the transcriptional stability of endogenous control genes using geNorm and NormFinder programs

Endogenous control genes are presumed housekeeping genes which are expected to have minimal expression fluctuation in comparison with other genes in a cell at different environmental conditions. However, in given conditions, their expression may vary considerably [31], [32]. Since there is no consensus for internal control in bacteria, there is the frequent need for the determination of internal control genes to normalize mRNA fractions in every study. Ginzinger [33] reported that such an effort requires the selection of presumed housekeeping genes with highly stable gene expressions at different experimental conditions; and high PCR efficiencies.

In order to determine a stable endogenous reference for gene expression experiments, four genes were chosen: (i) two genes encoding RNA polymerase subunits (the *rpoB* gene encoding bacterial β subunit of the RNA polymerase and *rpoD* gene encoding sigma factor (SigD protein) from the sigma-70 family); (ii) a gene involved in cell division and DNA replication (*dnaG* encoding the primase); and (iii) the *rrs* gene encoding the 16S rRNA (Table 1). All of the genes were selected from literatures [34], [35], [36] and their sequences are available in the strain's genome sequence with the EMBL/GenBank accession number CP000656. Their transcript levels were measured in all the sample conditions: pH 5.5, 6.5, 7.5; 0 M, 0.17 M, 0.5 M, 0.6 M and 1 M NaCl concentrations; and control, making nine in all, at different times of 0, 12, 24, 36 and 48 hours; correlating with the residual pyrene sampling analysis.

**Figure 2:**
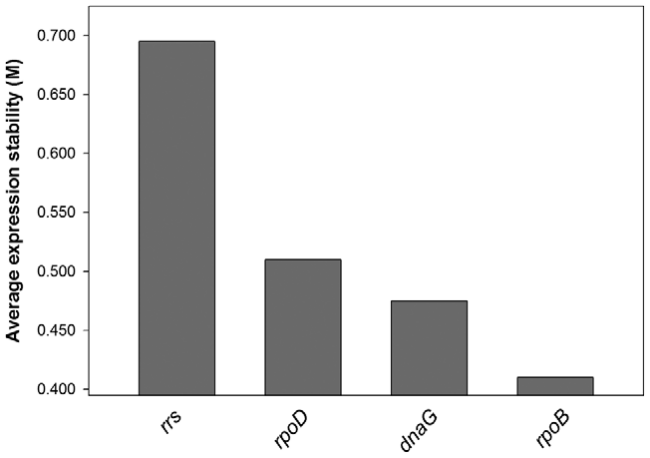
A chart indicating the value M of candidate reference genes. The M value refers to the average expression stability of reference genes during a stepwise exclusion of the least stable expressed reference gene. Starting from the least stable gene at the left, the genes are ranked according to increasing expression stability, ending with the two most stable genes on the right (*dnaG* and *rpoB*).

**Figure 3:**
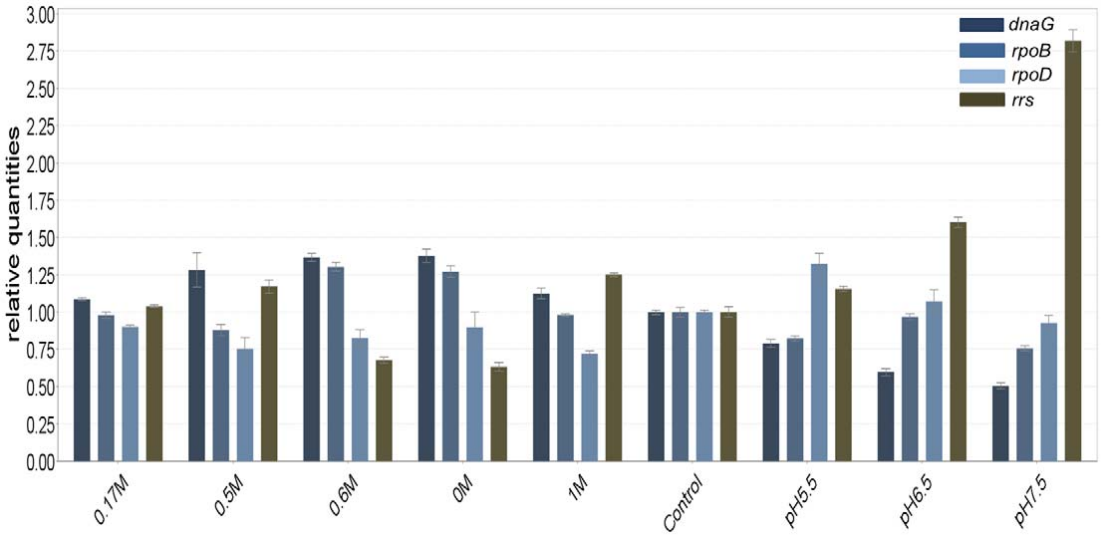
Histogram of cycle thresholds (C_T_) of the four presumed house-keeping genes. The transcript quantifications were determined in nine different conditions: pH 5.5, pH 6.5, pH 7.5, NaCl concentrations of 0 M, 0.17 M, 0.5 M, 0.6 M, 1 M and a control state of no pyrene induction. For each condition, C_T_ was measured from two independent cDNA; the means are represented in this histogram.

GeNorm calculates expression stability value (M) for each candidate gene based on pairwise comparisons of variability. Each gene is compared to every other gene to determine two genes with the least variation and the stability value is used to rank genes from the most stable to the least stable. The authors of the method give an M-value of 1.5 as a cut-off for suitability of an endogenous control gene [32] and all the genes used for this study were well below the cut-off value (Fig. 2). Gene expression levels of candidate endogenous control genes are displayed in Fig. 3. The most stably expressed gene identified by the geNorm software was *rpoB*.

NormFinder ranks a set of endogenous reference genes according to their expression stability in a given sample set and a given experimental design [37]. The C_T_ values for all the candidate endogenous reference genes were evaluated with the stability value of NormFinder (version 0.953). The stability values of the four candidate genes are shown on Table 2. The result also corroborated the geNorm result identifying the *rpoB* gene as the most stable reference gene in the nine sample conditions.

**Table 2:** Stability values of the candidate endogenous genes generated by the NormFinder program based on their threshold cycle (C_T_) values.

Genes	Stability value
*Rrs*	0.666
*rpoD*	0.274
*rpoB*	0.269
*dnaG*	0.559

Gene symbols represent: *rrs* (16S RNA ribosomal subunit: Mflv_ R0023), *rpoB* (DNA-directed RNA polymerase subunit: Mflv_5097), *rpoD* (RNA polymerase subunit, sigma-70 family: Mflv_4912), *dnaG* (Primase: Mflv_2722). The gene with the least stability value (*rpoB*: 0269), was identified as the most stable gene across all the sample conditions tested.

### Application of selected endogenous reference gene to pH and salinity changes on aromatic ring-cleaving dioxygenases study

**Figure 4:**
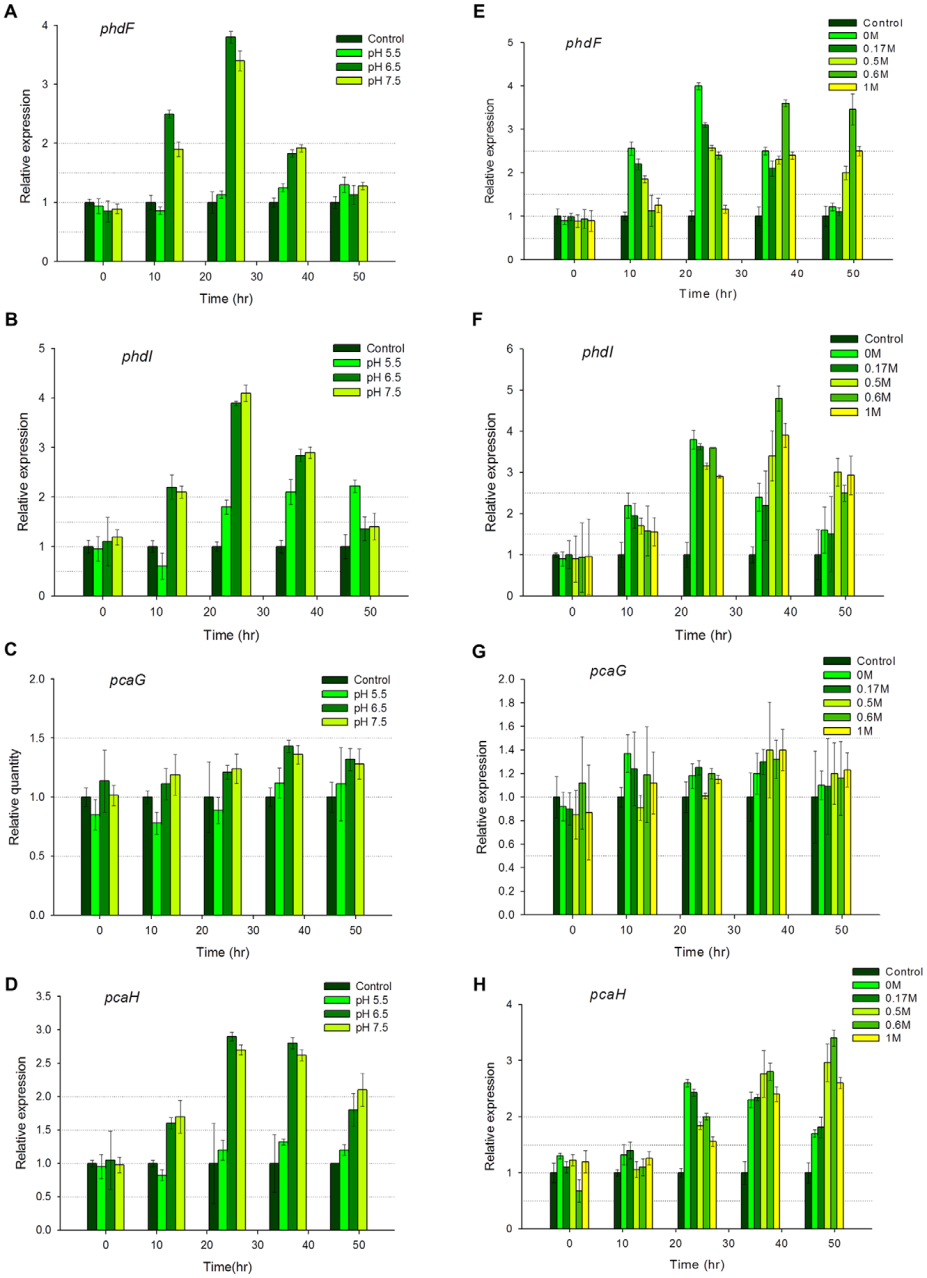
Relative expression levels of the ring-cleavage dioxygenase genes after induction. The graphs of quantified transcript levels after various pHs induction (A–D) and different NaCl concentrations induction (E–H). Gene expression is quantified using qRT-PCR and the comparative critical threshold (2^ΔΔCT^) method. The *rpoB* gene was used as the endogenous reference, while the expression in the control sample (without pyrene substrate) was used as the calibrator. Two independent experiments were performed. Vertical bars indicate means from standard deviations of triplicate qRT-PCR analyses.

The aim of the best endogenous gene search was to analyze the various gene expression levels of the aromatic ring-cleaving dioxygenase genes (*phdF, phdI, pcaG* and *pcaH*) in the different states of NaCl concentration and pH levels with accuracy. With the *rpoB* gene showing the least expression variation in the preliminary experiments, it was therefore used as an internal control to normalize the gene expression for further studies. The control sample, without any carbon source was used as a calibrator to calculate the relative expression levels. All genes were differently expressed in all the samples as expected. In the pH induced cells, a general upregulated expression was observed at pHs 6.5 and 7.5. Three out of the four genes studied, *pcaG, pcaH* and *phdF*, had their activities repressed in the pH 5.5 induced cells, with their highest expression values slightly above the calibrator (Fig. 4A, C and D). An exception was made of *phdI* with an expression activity level of above 2-fold in the pH 5.5 induced cells (Fig. 4B). At pHs 6.5 and 7.5, *phdF* and *pcaH* were highly expressed with *phdF* attaining an expression value of ∼4-fold after 24 hours of cultivation; while *pcaH* was ∼3-fold expressed at the 24th and 36th hour of induction. *pcaG* was lowly expressed in all induction conditions and this phenomenon was echoed in an earlier proteomic study [20] and in a similar pyrene degradation systems biology study using *Mycobacterium vanbaalenii* PYR1 [25].

Different NaCl concentration inductions of the cells generated various degrees of gene expression activities. *phdF* was ∼4-fold expressed at NaCl concentrations of 0 M and 0.17 M at the 12th and 24th hours (Fig. 4E). At the 36th and 48th hours, same gene was expressed above 2-fold in the 05 M, 0.6 M and 1 M induced cells. This observation may be as a result of late gene expression activity induced in the cells at high NaCl concentrations. Fig. 4F shows *phdI* expressed ∼5-fold at 0.6 M and above 1.5-fold in all the other induction conditions, suggesting a strong tolerance of the translated enzyme for high salt concentrations. *pcaG* was not significantly expressed as expected but activities were observed at all the NaCl concentration conditions tested, although they had no specific correlation (Fig. 4G). *pcaH* was significantly expressed in all the NaCl conditions tested, with its highest expression recorded above 3-fold in the 0.6 M induced cells (Fig. 4H).

Overall, the gene expression pattern observed across all the analyses showed a possibly delayed activity in the cells induced with high NaCl concentration compared to the cells induced with lesser NaCl concentrations.

## Discussion

The aims of this study were: (i) to quantify pyrene degradation in the different states of pH and salinity concentration; (ii) to acquire a validated endogenous gene reference for a gene transcript expression quantification study in *M.gilvum* PYR-GCK and (iii) to study the expression of several aromatic ring-cleaving dioxygenase genes in different states of pH and salinity concentrations.

We have successfully used the combined techniques of gas chromatography/ flame ionization detection and RT-qPCR to quantify cultural residual pyrene and identify aromatic ring cleaving dioxygenase genes differentially expressed in various pH states and salinity concentrations, respectively. The sample conditions: pHs 5.5, 6.5, 7.5, correspond to the pH changes encountered in acidic soils and oceans polluted with PAH compounds while the conditions of 0 M (0 g/L), 0.17 M (10 g/L), 0.5 M (29 g/L), 0.6 M (35 g/L) and 1 M (58 g/L) NaCl concentrations correspond to the salinity concentrations of the marine environment and some industrial waste effluents [13].

Pyrene (PAH) degradation can occur in various environmental conditions. The laboratory developed conditions were made to mimic these environmental conditions as much as possible. This study has shown the feasibility of pyrene degradation at different states of pH. With reports on ocean acidification [38], there is the possibility of pyrene degradation. There has been no report of highly acidified oceans (due to the carbonate buffering system) but in the weakly acidified states, pyrene degradation activities do occur, as shown by our residual pyrene and gene expression results. The slightly acidic nature may increase the pyrene degrading activity as a result of increased cell membrane permeability to pyrene substrates [11]. This knowledge of pyrene degradation activity may probably be more applicable to soils which undergo different rates of acidification as a result of PAH pollution.

Fluctuating salt concentrations may be detrimental to an environmental habitat that is not functionally equipped for it. The ocean with an approximate salinity concentration of 0.6 M (35 g/L) has been a culprit of PAH pollution in recent times as a result of off-shore drillings and crude oil tanker spills. *M.gilvum* PYR-GCK has shown exceptional adaptive ability to degrade pyrene at zero to 1 M salinity degrees, making it a good candidate for molecular study. A reduction in pH from 7.5 to 5.5 suppressed the genes' activities while the salinity increment strengthened their active expression. This halotolerant nature is believed to be as a result of the strain's original habitat of isolation, an environment heavily polluted with industrial effluents and its proximity to an estuary. Also, the salinity tolerance of the strain may be attributed to its relative's halotolerant characteristic acquired as a result of ectoine and hydroxyectoine osmolytes in their cells [14]. Applying the strain's bioremediation activity for waste water treatment however, may effectively occur at a slower rate compared to its activity in a more diluted wastewater. Likewise, it is highly suggested to neutralize any strongly acidic industrial effluent or polluted substrate, to a slightly acidic state for an effective bioremediation activity.

The qRT-PCR technique is an excellent research tool for accurately quantifying gene expression due to its combined qualities of specificity, sensitivity, speed and practical simplicity [39]. Normalization is a critical factor in reporting RT-PCR expression data, providing a necessary control for error associated with sample preparation. Normalization using endogenous control genes provides a means of controlling this error, provided the gene used is stably expressed across the entire sample under investigation [40]. The determination of an internal control preceded the qRT-PCR study as Ginzinger [33], Vandecasteele [31] and Vandesompele et al. [32] had suggested, in order to get an accurate quantification of the transcripts. There is no consensus for prokaryotic internal control genes unlike the eukaryotes with known house-keeping genes such as GADPH and beta actin genes. Therefore, the transcript level of four postulated house-keeping genes were quantified in nine conditions of pH and salinity changes. Most studies use the *rrs* gene for quantification studies but the 16S rRNA gene is not the best representative of the mRNA fraction since the rRNA molecules is about 95% of the whole RNA mass. This will evidently bring about a biased quantification of the expressed genes. Also, our quest for the best internal control gene revealed the highly unstable expression of the *rrs* gene in the experimental conditions (Fig. 2). Therefore, instead of using the classical *rrs* gene, we opted for *rpoB* gene which was averagely stable in all the conditions tested compared to all the other internal control gene candidates.

The qRT-PCR method was used to study the transcript levels of the four aromatic ring-cleaving dioxygenase gene activities in the *M.gilvum* PYR-GCK strain. This study has shown significant expression activity of the tested genes at the various conditions and a marked correlation has been observed between the residual pyrene analysis and the gene expression study. This is the first gene-expression research of this nature in pyrene degradation studies and more so in any pyrene degrading bacterial strain. Proteomic gene expression studies have been done in various bacterial strains. In the *Mycobacterium* genus, a study on *M. vanbaalenii* PYR-1 [25] showed the expression of these aromatic ring-cleaving dioxygenases during pyrene induction. The report showed an expression level of 3.4 fold in PhdI, pyrene-only expression of the PhdF protein, down regulated (0.5) protein expression of the PcaG and a 1.6 fold expression of the PcaH protein; all calibrated with a sorbitol-induced protein expression. An earlier proteome expression study on *M.gilvum* PYR-GCK [20], showed similar results with PhdF and PhdI solely expressed in the pyrene induced cells, the PcaG protein weakly upregulated in the pyrene induced cells and PcaH protein 2.9 fold up-regulated in the pyrene induced cells; all calibrated with a glucose-induced protein expression. Our research probably used a starving set of *M.gilvum* PYR-GCK strains, without any source of carbon or energy, in calibration. None of the previous studies were in any way based on the conditions listed in our research but instead their studies were all carried out at optimal conditions of pH 7.0 and 0 M salinity. This was the conditional basis of our calibration sample, equipped with null substrate for zero induction. The results we acquired from our study echoed a similar pattern with the protein expression studies, thereby validating our findings.

### Conclusion

In pyrene degradation, the critical step of ring fission is catalyzed by ring-cleaving dioxygenases. These enzymes, coded for by their respective genes, have to be highly functional for an effective activity. At various environmental conditions, pyrene degradation rates are affected either positively or negatively. From this study, we have proposed the use of halotolerant organisms, *M. gilvum* PYR-GCK inclusive, in bioremediation activities; and for a faster pyrene biodegradation rate, a neutralization of the substrate environment to pH 6.5 is suggested.
